# Association of Combining Diet and Physical Activity on Sarcopenia and Obesity in Elderly Koreans with Diabetes

**DOI:** 10.3390/nu16070964

**Published:** 2024-03-27

**Authors:** Sohye Kim, Soojeong Kim, Kyung Hee Hong

**Affiliations:** 1 Nutrition Care Services, Seoul National University of Bundang Hospital, Seongnam 13620, Republic of Korea; sohye76@daum.net; 2 Department of Health Administration, Dongseo University, Busan 47011, Republic of Korea; sjkim@dongseo.ac.kr; 3 Department of Food Science and Nutrition, Dongseo University, Busan 47011, Republic of Korea

**Keywords:** dietary factor, diabetic elderly, KNHANES, obesity, physical activity, sarcopenia

## Abstract

This study aimed to identify the combined factors of physical activity and diet associated with non-sarcopenic non-obese status in 1586 diabetic patients aged ≥65 years from the Korean National Health and Nutrition Examination Survey (2016 to 2019). Participants were categorized into non-sarcopenic non-obesity (NSNO), sarcopenia non-obesity (SNO), non-sarcopenic obesity (NSO), and sarcopenic obesity (SO) groups. NSNO had lower insulin, HOMA-IR, and triglycerides compared to NSO and SO. NSNO had lower perceived stress, higher nutrition education and dietary supplement intake. As assessed by the Korean Healthy Eating Index, NSNO scored higher total than SNO and SO, in breakfast and energy balance compared to SO, and in the adequacy of vegetables and meat/fish/egg/bean compared to SNO. NSNO had significantly higher energy and protein intake and physical activity, with BMI/waist circumference lower than NSO, SO, and comparable to SNO. Physical activity was positively associated with NSNO. Low Total KHEI score and protein intake level reduced the odds ratio (OR) of NSNO, particularly when physical activity was insufficient, with OR = 0.38 for KHEI Q1 and OR = 0.32 for protein T1. In conclusion, physical activity, diet quality, and protein intake are associated with NSNO prevalence in Korean elderly with diabetes, and energy balance through active intake and expenditure may be effective.

## 1. Introduction

Diabetes mellitus stands out as the most prevalent chronic metabolic disorder worldwide, exhibiting an increasing trend in prevalence among the elderly due to the extension of life expectancy [1,2]. As aging progresses, alterations in body composition manifest, characterized by a decline in muscle mass and strength coupled with an increase in body fat, become prominent due to decreased physical activity and metabolic rate. Consequently, these changes predispose individuals to the development of both sarcopenia and obesity [3]. The intricate interplay between sarcopenia and obesity mutually exacerbates their development, concurrently exerting adverse effects on glucose metabolism and insulin resistance. Excessive fat mass associated with aging fosters muscle insulin resistance and consequently leads to sarcopenia [4]. Given the pivotal role of maintaining muscle mass in glucose homeostasis [5], age-related sarcopenia contributes to impaired blood glucose regulation, increased insulin resistance, and the onset of diabetes [6]. Conversely, the reduction in muscle mass and strength leads to decreased energy expenditure, contributing to the promotion of adiposity and obesity [7], and in turn, obesity perpetuates a vicious cycle by increasing insulin resistance, thereby expediting the onset and progression of diabetes [8]. 

In the elderly, sarcopenia is not only a consequence but also a potential cause of diabetes, highlighting a bidirectional relationship between these two conditions [1,2]. Sarcopenia in the elderly has been reported to elevate the risk of diabetes by approximately two-fold [9], while conversely, the presence of diabetes increases the risk of sarcopenia by 1.5-fold [10]. In the elderly, diabetes and sarcopenia are intricately linked, resulting in negative outcomes such as increased cardiovascular disease risk, overall health decline, elevated fall risk, frailty, diminished overall quality of life, chronic diabetes complications, and mortality [2]. Therefore, in the elderly with diabetes, it is crucial to manage both preserving skeletal muscle mass and strength and preventing excessive fat accumulation. Maintaining a state where both sarcopenia and obesity are absent becomes vital for improving blood glucose control and preventing complications.

Lifestyle modification can be an effective strategy to delay and ameliorate age-related sarcopenia and obesity in diabetes [1,2]. Long-term lifestyle interventions focusing on diet and physical activity have been shown to delay disability and extend healthy life expectancy in the elderly with diabetes [11]. Despite diabetic patients facing a higher risk of nutritional deficiencies, expanded nutritional counseling has proven effective in significantly reducing the incidence of sarcopenia [12]. Diet intervention emerges as a crucial strategy not only for managing muscle mass and strength in the elderly but also for preventing diseases associated with malnutrition, showing positive effects [8]. Moreover, high-quality diets rich in antioxidants have been inversely associated with fasting glucose, abdominal obesity, and sarcopenia in diabetic patients [13], and higher dietary quality was associated with a reduced risk of metabolic syndrome [14]. Furthermore, regular exercise in older adults with diabetes is widely used to prevent muscle breakdown and decline in physical function associated with the aging process, contributing to improved glycemic control, insulin resistance, body fat, lipid profiles, and cardiovascular health [1,2]. Therefore, a combination of exercise and a high-quality balanced diet appears to be the safest and most promising strategy for managing both sarcopenia and obesity in the elderly with diabetes. Nonetheless, overall, self-diet management has been found to be inadequate in Korean type 2 diabetes patients, with self-management ability declining as the duration of illness increases [15]. Additionally, considering that the impact of nutrients on health outcomes varies with age, proposed clinical nutrition intervention for elderly diabetes patients focus on age-specific dietary strategies, shifting from strict restrictions for obesity to preventing frailty and sarcopenia [16]. This suggests the need for efficient and differentiated management to prevent complications and improve quality of life in the elderly with diabetes.

However, research investigating health-related dietary factors based on sarcopenia and obesity status in Korean elderly with diabetes is limited. Studies using the Korean National Health and Nutrition Examination Survey (KNHANES) 2008–2011 data have reported the separate impacts of physical activity and protein intake [17], and the combined effects of energy intake and physical activity [18], on sarcopenia, obesity, and sarcopenic obesity in the elderly. However, there is a lack of recent studies investigating the association between the combined factors of physical activity and diet with non-sarcopenic non-obese status in Korean elderly with diabetes. Therefore, in this study, we aim to identify dietary factors associated with non-sarcopenic non-obese status in the elderly with diabetes and evaluate the combined factors of physical activity, diet quality, and protein intake level in relation to this status, using the KNHANES 2016–2019, the most recent data available on muscle strength, a diagnostic criterion for sarcopenia. 

## 2. Materials and Methods

### 2.1. Data Source and Study Population

This study utilized the raw data from KNHANES, a representative dataset for the Korean general population extensively employed as a national statistic. The focus was on diabetic patients aged 65 years and older, encompassing 1880 participants from the 6th (2016–2018) and 7th (2019) KNHANES. Diabetes was diagnosed based on criteria such as fasting blood glucose levels of 126 mg/dL or higher, a physician’s diagnosis, use of blood glucose-lowering medications or insulin injections, or HbA_1_c level of 6.5% or higher. After excluding participants without measurements for grip strength and waist circumference, those lacking dietary survey data, and those who did not undergo blood tests, the final analysis included a total of 1586 elderly diabetic patients.

### 2.2. Definition of Sarcopenia and Obesity

In this study, sarcopenia was diagnosed based on muscle strength, with handgrip strength (HGS) measurements from the KNHANES. A definition of sarcopenia emphasizing muscle impairment in terms of strength rather than mass was adopted [3]. Notably, a more robust relationship with mortality has been observed when diagnosing sarcopenia using qualitative muscle measurements such as HGS, rather than muscle mass [19]. HGS was measured with a digital grip strength dynamometer (TKK 5401 Grip-D; Takei, Niigata, Japan). The maximum value from the dominant hand, primarily used for three measurements (2016–2018), or the maximum value measured in 2019, was considered. Following the Asian Working Group for Sarcopenia (AWGS) 2019 criteria [20], individuals with HGS below <28 kg for men and <18 kg for women were diagnosed with low HGS, classifying them as sarcopenia. Obesity diagnosis relied on waist circumference, with the Korean Society for the Study of Obesity (KSSO) cutoffs defining abdominal obesity as ≥90 cm for men and ≥85 cm for women [21]. Participants were classified into four groups based on the combinations of sarcopenia and obesity definitions mentioned above: Non-Sarcopenic Non-Obesity (NSNO), Sarcopenia Non-Obesity (SNO), Non-Sarcopenic Obesity (NSO), and Sarcopenic Obesity (SO).

### 2.3. Health and Dietary Behavioral Characteristics

Health behavioral variables, such as smoking, alcohol consumption, perceived stress, and physical activity, were identified. Alcohol consumption was defined as having consumed alcohol at least once a month in the past year, and perceived stress was defined as feeling a significant amount or a lot of stress in daily life. Recommended physical activity was defined as vigorous activity at least 3 times per week for 20 min or more each time, or moderate activity at least 5 times per week for 30 min or more each time, according to the criteria for ‘Aerobic Physical Activity and Strength Training Practice Rates’ from KNHANES [18]. Dietary behavioral variables were investigated, including nutrition education experience, frequency of eating out, food security, and the use of dietary supplements. Nutritional education experience was defined as having received nutritional education or counseling within the past year. Dietary supplement intake was defined as having taken dietary supplements regularly for at least two weeks within the past year. 

### 2.4. Diet Quality (KHEI) 

The study assessed diet quality using the Korean Healthy Eating Index (KHEI) scores developed by the Korean Centers for Disease Control and Prevention (KCDC). The KHEI comprises 14 components aligned with healthy eating habits for Koreans. Specifically, it includes eight items assessing the adequacy of daily intake for a quality diet (having breakfast, intake of mixed grains, fruits, fresh fruits, vegetables, vegetables excluding kimchi and pickles, meat/fish/eggs/beans, and milk/milk products), three items for moderation elements that should be limited (percentage of energy intake from saturated fatty acids, sweets and beverages, and sodium), and three items for the balance of energy intake (percentage of energy intake from carbohydrate, fat, and adequate energy intake). Each item is scored on a scale of 5 or 10 points, resulting in a total of 100 points (Supplementary Table S1).

### 2.5. Energy and Nutrient Intake 

Variables related to energy and nutrient intake were evaluated using data from dietary intake surveys collected using the 24-h recall method in KNHANES. The analysis included the assessment of daily intake of energy and macronutrients (carbohydrates, protein, and fat), energy and protein per body weight (kg), and dietary fiber. Additionally, the study examined the percentage of energy intake from carbohydrates, protein, and fat, as well as the distribution of energy intake (%) by meals, including breakfast, lunch, dinner, and snacks.

### 2.6. Statistical Analyses

The KNHANES raw data underwent analysis, considering the complex sample design (strata, clusters, and weights) due to multistage clustered sampling. SPSS Statistics ver. 28.0 (IBM SPSS, Armonk, NY, USA) was employed for all analyses. To assess the distribution of general characteristics, biochemical measures, health and dietary behaviors, KHEI, and energy and nutrient intake among various groups of study participants, complex sample cross-tabulation and the Complex Samples General Linear Model (CSGLM) procedure were employed. Results from the complex sample cross-tabulation were presented with unweighted frequency and population-estimated percentage (weighted %). Pearson chi-square statistics were used to evaluate group differences. CSGLM analysis results were presented as estimated means and standard errors, and differences in means were assessed using Wald F statistics. The NSNO group served as the reference group, and the LSD method was utilized to test mean differences. For assessing the association between dietary factors and sarcopenia and obesity status, multivariate logistic regression analysis was conducted to derive odds ratios (ORs) and 95% confidence intervals (CIs). The statistical significance level was set at *p* < 0.05. 

## 3. Results

### 3.1. General Characteristics

Among the 1586 participants in this study, 418 (26.8%) belonged to the NSNO group, 275 (18.2%) to the SNO group, 579 (34.9%) to the NSO group, and 314 (20.1%) to the SO group. The general characteristics of the participants are presented in Table 1. Both education level and employment rate were highest in the NSNO group compared to the other groups, while the SO group had the lowest values. House income was also higher in the NSNO group, with the proportion of middle-high or high levels with 30.2%, comparable to the NSO group (29.2%). BMI was significantly lower in the NSNO group compared to both the NSO and SO groups, and slightly higher than the SNO.

### 3.2. Clinical Characteristics

Table 2 presents differences in clinical characteristics, using the NSNO group as the reference. Compared to the NSNO group, insulin levels were significantly higher in the NSO and SO groups, HOMA-IR was also significantly higher in the NSO group, with non-significant yet increasing trend in the SO group. Moreover, serum triglyceride concentrations were significantly higher in the NSO and SO groups compared to the NSNO group. Fasting blood glucose, HbA_1_c, systolic and diastolic blood pressure, total cholesterol, HDL-cholesterol, and LDL-cholesterol concentrations did not exhibit significant differences among the groups. 

### 3.3. Health and Dietary Behavioral Characteristics

Table 3 presents the health and dietary-related behavioral characteristics of the participants. The NSNO group had a higher proportion of alcohol consumption compared to the other groups, and it reported the lowest percentage of perceiving stress. Additionally, the NSNO group showed higher frequencies of eating out and experiences with nutritional education compared to the SNO and SO groups, along with the highest proportion of dietary supplement intake among the four groups. The percentage of participants engaging in physical activity was significantly higher in the NSNO group (41.1%) compared to the SNO (22.8%), NSO (29.3%), and SO (16.8%) groups.

### 3.4. Diet Quality (KHEI)

Adjusted for age and gender, differences in KHEI scores among the groups were compared using the NSNO group as the reference, and the results are presented in Table 4. The NSNO group had a significantly higher total KHEI score compared to the SNO and SO groups. The NSNO group showed significantly higher adequacy scores for total vegetables, vegetables excluding kimchi and pickles, meat/fish/eggs/beans, and moderation scores for sodium intake compared to the SNO group. Additionally, the NSNO group had higher adequacy scores for breakfast and balance scores for total energy intake compared to the SO group. No statistically significant differences in KHEI scores were observed between the NSNO and NSO groups. 

### 3.5. Energy and Nutrient Intake

In Table 5, we present comparisons of energy and nutrient intake adjusted for age and gender among groups, with the NSNO group as the reference. Total energy intake was significantly higher in the NSNO group compared to the SNO group, and although not statistically significant, it exhibited higher level compared to the SO group. Moreover, when comparing energy intake per kg body weight (B.W.), the NSNO group (28.3 kcal/kg B.W.) was significantly higher than the NSO and SO groups (14%, 20% higher, respectively). Additionally, when comparing protein intake per kg B.W., the NSNO group (0.96 g/kg B.W.) demonstrated the highest significant difference compared to the SNO, NSO, and SO groups (11.6%, 17.1%, 23.1% higher, respectively). Daily protein and dietary fiber intake were higher in the NSNO group than the SNO group. The percentage of energy from carbohydrates was consistently higher than the Korean dietary reference intake range of 55–65% across all groups, with no significant differences observed. The proportions of energy from protein, fat, and the distribution of energy by meals (breakfast, lunch, dinner, and snacks) did not show statistically significant differences among the groups.

### 3.6. Association of the Combination of KHEI Score, Protein Intake, and Physical Activity with Non-Sarcopenic Non-Obese Status

In Table 6, we presented the prevalence of NSNO based on the combination of physical activity and KHEI score, as well as the combination of physical activity and protein intake level. After adjusting for confounding factors (Model 3), the prevalence of NSNO significantly decreased when physical activity was insufficient compared to the recommended level at the same KHEI score quartile, and a significant association between KHEI score and NSNO was observed when physical activity was insufficient. Thus, when physical activity is insufficient, the odds ratios (ORs) for NSNO decreased to 0.60 (CI: 0.36–0.99) in KHEI Q4, further significantly decreasing to 0.38 (CI: 0.22–0.66) in Q3, 0.51 (CI: 0.28–0.92) in Q2, and 0.38 (CI: 0.21–0.69) in Q1. However, when physical activity was at the recommended level, a decreasing trend in NSNO prevalence with lower KHEI score quartiles was observed in all models, but was not statistically significant. 

In the combined effect analysis of physical activity and protein intake level, we also observed a significant decrease in NSNO prevalence at the same protein intake tertile when physical activity was insufficient compared to when it was at the recommended level. In model 3, protein intake level showed and association with NSNO when physical activity was insufficient, resulting in ORs of 0.43 (CI: 0.22–0.84) at T2 and 0.32 (CI: 0.17–0.61) at T1, decreasing with each tertile. When physical activity was at the recommended level, there was a trend toward lower NSNO prevalence as protein intake tertiles decreased, but this was not statistically significant. In model 1 and model 2, the ORs decreased to 0.41 (CI: 0.22–0.77) and 0.50 (CI: 0.26–0.97) at T1, respectively, even at the recommended physical activity level.

### 3.7. Association of KHEI and Protein Intake with Sarcopenia and Obesity Status

The ORs for SNO, NSO, and SO according to the total KHEI score and the level of protein intake are presented in Table 7. In model 4, after adjusting for all possible confounding factors, an association was observed between KHEI score and SO, with odds increasing by 2.0-fold in Q1 (95% CI: 1.03–3.87). In model 3, the odds for SNO also increased by 1.93-fold in Q1 (95% CI: 1.10–3.38). Regarding protein intake level, in model 4, prevalence of NSO increased at T1 (OR: 2.15, 95% CI: 1.34–3.46). For SO, prevalence increased as protein intake tertile decreased, with ORs at T2 (OR: 1.88, 95% CI: 1.02–3.48) and T1 (OR: 2.99, 95% CI: 1.60–5.57). In model 3, odds for SNO also increased at T1 (OR: 1.96, 95% CI: 1.11–3.47).

## 4. Discussion

This study investigated dietary factors associated with non-sarcopenic non-obese status and evaluated the combined effect of physical activity and diet to this status among elderly with diabetes who participated in the KNHANES from 2016 to 2019.

The research findings revealed that the NSNO group exhibited higher levels of education and household income compared to those with sarcopenia or obesity, which aligns with similar results observed in a study of Korean elderly [22]. Furthermore, social status was identified as an important risk factor for both sarcopenia and diabetes [2]. These findings indicate that in diabetic elderly, a higher socioeconomic status is associated with a higher likelihood of maintaining a non-sarcopenic non-obese status.

The present study showed that non-sarcopenic non-obese status is associated with lower fasting insulin levels and HOMA-IR compared to obesity or sarcopenic obesity, indicating a lower state of insulin resistance. Similar to our study, in Korean elderly, non-sarcopenic non-obese individuals had lower insulin levels and HOMA-IR compared to those with obesity, with or without sarcopenia [23], as well as in sarcopenic elderly, HOMA-IR was higher in both obese and non-obese [17,24]. Muscle strength showed an inverse association with insulin levels, insulin resistance, and type 2 diabetes [25], and sarcopenia demonstrated a significant association with insulin resistance, particularly in the elderly [9]. These results collectively highlight that both obesity and sarcopenia are factors associated with insulin resistance. Age-related sarcopenia, obesity, and insulin resistance suggests a complex interplay with mutually detrimental effects. The reduction in muscle mass and strength in sarcopenia leads to decreased energy expenditure, contributing to increased body fat and obesity [7]. Sarcopenia appears intricately linked to glucose metabolism, emphasizing the crucial role of maintaining muscle mass in glucose homeostasis [5]. Thus, age-related changes of skeletal muscles related to aging lead to impaired blood glucose control, increased insulin resistance, and the development of diabetes [6]. Moreover, poor glycemic control accelerates skeletal muscle loss and structural changes, initiating a vicious cycle of age-related muscle alterations in diabetic patients [6]. Insulin resistance correlates independently with muscle strength, and elderly diabetic patients exhibit accelerated decline in leg strength and quality [4]. Obesity, on the other hand, through insulin resistance, may cause muscle loss and functional impairment [4], which can accelerate the progressive decline in glucose and lipid metabolism in skeletal muscles, thereby expediting the progression of diabetes and entering a vicious cycle [8]. Sarcopenic obesity in diabetic patients is associated with serious complications [8]. Ultimately, the interplay between muscle loss and fat accumulation exacerbates metabolic dysfunction through insulin resistance. Conversely, insufficient nutrient supply to muscle tissue due to insulin resistance leads to muscle atrophy and decline. This, in turn, contributes to a vicious cycle where sarcopenia exacerbates glycemic dysregulation [5,8]. These results indicate the importance of maintaining a non-sarcopenic non-obese status in diabetic patients is crucial to prevent insulin resistance escalation and impairments in blood glucose regulation, contributing to effective diabetes management and complications prevention. In addition, the study findings revealed that non-sarcopenic non-obesity exhibited lower serum triglyceride concentrations compared to obesity or sarcopenic obesity. Similarly, previous research on Korean elderly showed that non-sarcopenic non-obesity had lower serum triglycerides compared to obesity with or without sarcopenia [23,26], as well as a higher proportion of individuals with high triglycerides [18]. Additionally, sarcopenia was associated with elevated serum triglycerides in both obese and non-obese individuals [24]. These results also suggest the importance of maintaining a non-sarcopenic non-obesity status for blood lipid management. 

The NSNO group in this study exhibited the lowest perceived stress levels, and they reported more experiences with nutrition education and dietary supplement intake compared to patients with or without obesity, indicating better mental health and dietary behaviors in this group. On the other hand, despite having higher frequencies of eating out and alcohol consumption, they appeared to have a more flexible dietary lifestyle compared to diabetic elderly with sarcopenia or obesity. Diverse and complex factors were reported to influence the health-related quality of life in diabetic elderly in Korea, particularly with a significant correlation to perceived stress [27]. In type 2 diabetes, longer disease duration was associated with lower self-management levels, emphasizing the need for effective and sustained management to prevent diabetes complications [15]. Therefore, it appears that stress management, health-related dietary behaviors, and quality of life in diabetic elderly are associated with maintaining a non-sarcopenic non-obesity status.

Moreover, in the present study, the proportion of physical activity was significantly higher in the NSNO group compared to the other three groups. Physical activity is considered an important and potentially modifiable risk factor for both sarcopenia and diabetes [2], and it has been reported to be significantly correlated with the health-related quality of life in Korean diabetic elderly [27]. In diabetes, insufficient physical activity leads to skeletal muscle impairment and loss, subsequent visceral fat accumulation, events that are involved in the pathophysiology of glucose metabolism disorders, insulin resistance, impaired blood glucose control, and ultimately, type 2 diabetes [2,16]. Although no studies specifically address elderly individuals with diabetes, it has been reported that non-sarcopenic non-obesity had the highest prevalence of sufficient physical activity compared to sarcopenia or obesity, or both, in middle-aged [22] and older adults [18] in Korea. Additionally, physically active older adults exhibited higher lean mass, lower fat mass [28], and a decreased risk of sarcopenia with higher muscle strength [10,29]. Conversely, in diabetic elderly, less physical activity was negatively associated with sarcopenia, obesity, and sarcopenic obesity [18,30]. These results underscore the critical significance of adequate physical activity in diabetic elderly, as it proves to be effective in preventing and ameliorating both sarcopenia and obesity, thus playing a crucial role in addressing metabolic disorders and preventing complications in diabetes. 

The research findings indicate that the dietary quality of the NSNO group was better than SNO and NSO groups, including the total KHEI score, particularly in the adequacy of breakfast, vegetables, and meat/fish/eggs/beans, as well as the balance of total energy. Therefore, diabetic elderly with non-sarcopenic non-obesity status demonstrated a diet characterized by regularity, diverse food intake, and optimal energy intake. A study using KNHANES data revealed that type 2 diabetes aged 30 and older overall exhibit poor dietary management. Moreover, it was found that both dietary management and proper blood glucose control decrease, particularly as the duration of illness increases [15], indicating the importance of maintaining a high-quality diet for effective diabetes management. Similar to our research findings, when comparing the quality of diet using KHEI scores, non-sarcopenic non-obesity showed higher scores in total, adequacy, and energy balance than sarcopenia, obesity, or both in middle-aged and elderly Koreans [22]. Additionally, a study comparing diet quality using the Diet Quality Index for Koreans found that individuals without sarcopenia had an overall better diet quality than those with sarcopenia, and this difference in diet quality was particularly pronounced in Koreans aged 75 and above with a BMI of 23 kg/m^2^ or higher [31]. Furthermore, studies in Canada [28] and Spain [30], reported associations between the quality of a healthy diet in the elderly and higher lean mass, lower fat mass. These results demonstrate that maintaining a good diet quality is a crucial factor in preventing and ameliorating sarcopenia and obesity. Moreover, in diabetic patients, a diet rich in antioxidants nutrients showed an inverse relationship with fasting glucose, waist circumference, and sarcopenia [13], suggesting the association of diet quality with blood glucose control, abdominal obesity, and sarcopenia management. Additionally, in Korean adults, a diet metabolically healthy individuals had higher total scores in KHEI, adequacy scores for breakfast, and fruit intake, and higher KHEI scores were reported to be associated with a decreased risk of metabolic syndrome [14]. On the other hand, a study evaluating the quality of Korean adult diets using a modified KHEI that includes a Korean balanced diet revealed an inverse association between diet quality and abdominal obesity, whereas the modified KHEI for Western-style diets did not show such an association [32], emphasizing the importance of Korean-style balanced diets.

The research findings reveal that the NSNO group exhibited a significantly higher energy intake compared to other groups. Not only was their daily intake higher than that of the SNO group, but the per-unit body weight intake, at 28.3 kcal/kg B.W., was also higher compared to the NSO and SO groups, notably exceeding the intake of the SO group by approximately 20%. Although limited research has examined the effect of energy intake on sarcopenia and obesity in elderly diabetic patients, studies using KNHANES data have reported that non-sarcopenic non-obese older adults had the highest energy intake [26], and the highest proportion of energy intake above estimated energy requirement [18] compared with older adults with sarcopenia, obesity, and sarcopenic obesity. Moreover, an elevated OR for sarcopenic obesity was observed when energy intake was less than the recommended level [24], and the OR for sarcopenic obesity per 100 kcal increments of total energy intake was inversely associated [33] in Korean elderly. Therefore, ensuring sufficient energy intake is recommended for elderly diabetic patients, excluding those pathologically obese, to reduce and prevent the occurrence of sarcopenia [34]. The guidelines from the European Society of Clinical Nutrition and Metabolism recommend an energy intake of approximately 30 kcal/kg B.W. per day for elderly diabetic individuals, although energy intake should be adjusted according to individual conditions [28]. Comparatively, the observed levels of daily energy intake in elderly diabetic individuals with sarcopenia or obesity in this study (ranging from 23.6 to 26.9 kcal/kg B.W.) appeared insufficient. Particularly concerning were those with sarcopenic obesity, whose daily energy intake of 23.6 kcal/kg B.W. was even lower than 1 kcal/kg B.W./hour, underscoring the importance of optimizing energy intake in diabetic elderly. Additionally, the research findings indicate that the NSNO group not only had higher energy intake but was also engaged in more active physical activity, exhibiting lower BMI and waist circumference. Considering these results, non-sarcopenic non-obese diabetic elderly appear to maintain energy balance through active engagement in both energy intake and expenditure. In the Korean elderly, those who consume energy above the estimated energy requirement while simultaneously meeting the recommended level of physical activity showed significantly reduced risk for sarcopenia (OR: 0.44) and sarcopenic obesity (OR: 0.32) [18], supporting these outcomes. Furthermore, meta-analysis results suggest that restricting energy intake in the elderly leads to a decrease in body fat but also induces muscle loss, with muscle loss prevention observed only when exercise is combined [35]. Therefore, these findings underscore the importance of a combined approach involving adequate energy intake and sufficient physical activity to prevent muscle loss while maintaining appropriate body fat levels in the elderly.

Moreover, we also found that the NSNO group had significantly higher protein intake. When comparing protein intake (g/kg B.W.) with SNO, NSO, and SO, it was 11.6%, 17.1%, and 23.1% higher, respectively. Similarly, in a study of elderly using KNHANES data from 2008 to 2011, the NSNO group exhibited higher protein intake compared to sarcopenia, obesity, and sarcopenic obesity [26]. These results suggest that sufficient protein intake in the elderly is associated with preventing muscle loss and maintaining appropriate body fat levels. Aging blunts the response of muscle protein synthesis to anabolic stimuli, necessitating older adults to consume more dietary protein to maintain muscle mass than what is recommended for young adults [36,37]. Therefore, in older adults, adequate protein intake can be a successful strategy to combat diabetes and sarcopenia. Adequate dietary protein not only helps maintain muscle mass but also promotes body fat regulation and has positive effects on insulin sensitivity and blood glucose control, aiding in diabetes management [1,8]. Previous studies have reported associations between protein intake and sarcopenia in type 2 diabetes [34]. Additionally, low protein intake in individuals aged 65 and older correlated with higher mortality risk, especially in those 75 and older with diabetes [16]. Therefore, for elderly with diabetes, sufficient protein intake is crucial to reduce the risk of sarcopenia and improve glucose metabolism [34]. While adjustments should be made based on individual conditions, it is recommended that older adults consume at least 1.0 g/kg of protein per day, exceeding the recommended level for healthy young adults (0.8 g/kg B.W./day). For the maintenance and increase of lean mass, 1.0–1.2 g/kg B.W. is suggested, with 1.2–1.5 g/kg B.W. recommended for elderly with chronic conditions such as diabetes and sarcopenia obesity [8,36,38]. In this study, it can be noted as a concern that overall protein intake in elderly Korean individuals with diabetes was inadequate. Even in the NSNO group, which had the highest intake, the protein intake was 0.96 g/kg B.W., below the minimum recommended level of 1.0 g/kg. Particularly, the protein intake of diabetic elderly with SO was 0.78 g/kg B.W., lower than 0.8 g/kg B.W. This suggests that sufficient protein intake should be emphasized in Korean diabetic elderly to prevent muscle loss and maintain appropriate body fat. Additionally, the observed low energy intake in individuals with obesity with or without sarcopenia is presumed to be due to their adoption of energy-restricted diets for weight management; however, it has been suggested that low-calorie diets in the elderly are not effective for treating sarcopenic obesity, even when combined with high-protein [37]. In a previous KNHANES-based study, non-sarcopenic non-obese older adults had the highest proportion meeting both energy and protein requirements, associating with decreased sarcopenia odds [18]. Furthermore, meta-analyses have also shown that even with adequate protein intake in the elderly, insufficient energy intake can lead to muscle loss [35]. Thus, it is emphasized that for the management of sarcopenia and obesity in elderly diabetic individuals, both energy and protein intake should be sufficient concurrently. In this study, it is noted that in Korean elderly with diabetes, while protein intake is insufficient, there is an overall excessive carbohydrate contribution to energy intake (approximately 70–71%), which is pointed out as a concern, as it surpasses the KDRI of 55–65%. Previous research indicated that irrespective of sarcopenia and obesity status, elderly Koreans tend to consume over 75% of their energy from carbohydrates [18], particularly when overconsuming carbohydrate-rich foods such as wheat, leading to a 2.1-fold increase in the prevalence of sarcopenic obesity [39]. In addition, a study of middle-aged Koreans reported that the proportion of energy from carbohydrates, including refined grains such as white rice, serves as a determinant for metabolic syndrome. Moreover, higher proportion of energy from carbohydrates were associated with lower consumption of meat, fish, vegetables, fruits, milk, and dairy products, while kimchi consumption was higher [40]. These findings suggest that a carbohydrate-dominant dietary pattern may lead to a monotonous eating habit with limited food diversity, posing a risk of metabolic disorders in the elderly. The impact of nutrients on health outcomes varies with age; therefore, dietary strategies for elderly diabetic patients are suggested to shift from strict dietary restrictions to a diet-oriented perspective for the prevention of sarcopenia and frailty as they age. To achieve this, optimal energy intake and sufficient protein intake (1.0–1.5 kg/kg B.W.) are recommended. Additionally, a healthy dietary pattern promoting high diversity, including a substantial amount of vegetables and fish, and considering the diet-related quality of life, is proposed [16]. Therefore, it is suggested that efforts in managing diet and physical activity are crucial to enhancing the quality of life for elderly individuals with diabetes and extending healthy life expectancy.

In this study, when examining the relationship between dietary quality, protein intake levels, physical activity, and non-sarcopenic non-obese status in elderly with diabetes, physical activity emerged as the most significant determinant. When physical activity was insufficient, the ORs for non-sarcopenic non-obese status remained low, even in the highest quartile of KHEI Q4, indicating that inadequate physical activity, regardless of dietary quality, was associated with a reduced likelihood of non-sarcopenic non-obese status. Additionally, a lower KHEI score during inadequate physical activity was significantly associated with reduced odds of non-sarcopenic non-obese status, indicating an association between dietary quality and this status, especially in the context of insufficient physical activity. Therefore, these findings suggest that to maintain non-sarcopenic non-obese status in elderly individuals with diabetes, both physical activity and ensuring a high-quality diet should be pursued in combination. Moreover, protein intake levels also demonstrated an association with non-sarcopenic non-obese status. Lower protein intake levels were associated with a reduced likelihood, especially during insufficient physical activity, with ORs of 0.43 for T2 and 0.32 for T1. Thus, these results highlight the importance of a combination of attention to physical activity and adequate protein intake to maintain non-sarcopenic non-obese status in elderly with diabetes. Appropriate physical activity and a high-quality diet are effective countermeasures in the treatment of elderly individuals with diabetes, based on their bidirectional relationship [1]. A meta-analysis demonstrated that combining aerobic exercise and dietary therapy had a significant positive impact on diabetes, improving blood glucose, insulin, and HbA_1_c, along with BMI, waist circumference, blood pressure, total cholesterol, and triglycerides [41]. While energy restriction for weight loss in older adults is effective in reducing body weight and fat mass, it may also lead to a decrease in skeletal muscle mass. Therefore, for elderly with diabetes aiming for weight control, sufficient protein intake, especially when combined with exercise, can help prevent sarcopenia [37]. Studies of older patients with type 2 diabetes have shown that exercise combined with adequate protein intake is effective in preserving muscle mass; however, when protein intake is insufficient, exercise is shown to lead to a reduction in muscle mass [34], emphasizing the importance of combining adequate protein intake with physical activity in older adults with diabetes. On the other hand, we found that a low KHEI score is associated with an increased risk of sarcopenia with or without obesity, particularly in Q1, where the odds of sarcopenic obesity increase 2-fold. Additionally, low protein intake levels were not only associated with sarcopenia with or without obesity but also increased the odds of non-sarcopenic obesity. In T1, the odds of obesity increased by 2.15-fold, and the odds of sarcopenic obesity increased by 2.99-fold. Therefore, the importance of high dietary quality and sufficient protein intake in preventing sarcopenia and obesity in the elderly with diabetes, contributing to effective diabetes management, has been reaffirmed. Collectively, it is suggested that a high-quality diet ensuring energy balance and adequate intake of diverse foods, along with a protein intake of at least 1.2 g/kg B.W./day and concurrent physical activity, has the most positive impact on simultaneously managing sarcopenia and obesity in the elderly with diabetes.

This study has several limitations. First, being an analysis of KNHANES data with a cross-sectional design, it cannot establish causal relationships. Second, dietary data were collected using the 24-h recall method, which may not fully represent participants’ usual dietary habits. Third, the lack of a consensus definition for sarcopenia due to diverse definitions of muscle loss contributes to limitations. KNHANES 2016–2019 did not measure muscle mass, and muscle strength, assessed using grip strength, was used as the basis for diagnosing sarcopenia in this study. Fourth, the recommended levels of physical activity have not been validated, which limits the assessment of the association between physical activity and non-sarcopenic non-obese status. Finally, detailed factors related to diabetes, such as classification into type 1 or type 2 diabetes, or experiences of hypoglycemia, were not identified in KNHANES. Nevertheless, this study is valuable for its analysis of the combined effects of physical activity, diet quality, and protein intake in relation to sarcopenia and obesity status in Korean diabetic elderly, based on national big data.

## 5. Conclusions

In conclusion, this research suggests the necessity of maintaining an energy balance through active engagement in both energy intake and expenditure, achieved by simultaneous optimal energy intake and physical activity, to attain a non-sarcopenic non-obese status in elderly with diabetes. Additionally, we recommend sufficient protein intake of at least 1.2 g/kg B.W./day and advocate for a high-quality diet by transitioning from a simplistic, carbohydrate-dominant diet to diverse food consumption. This approach is proposed as a crucial factor in ameliorating sarcopenia and obesity simultaneously in elderly with diabetes, improving insulin resistance, and ensuring the prevention of complications and overall health assurance. The findings of this study provide valuable insights for planning dietary interventions, emphasizing the preservation of muscle mass and prevention of obesity, which is crucial for extending healthy life expectancy and maintaining a high quality of life in the elderly with diabetes. In future research, it is necessary to further stratify study subjects based on detailed factors related to diabetes classification and other factors associated with diabetes. Additionally, intervention studies are needed that differentiate physical activity into aerobic exercise and strength training, varying the intensity levels, and also stratifying protein intake levels for effective intervention.

## Figures and Tables

**Table 1:** General characteristics of subjects.

Variables	NSNO	SNO	NSO	SO	*p*-Value ^2^
Total	418 (26.8) ^1^	275 (18.2)	579 (34.9)	314 (20.1)	
Age	71.2 ± 0.2 ^3^	75.6 ± 0.3 ***	72.5 ± 0.3 ***	75.4 ± 0.3 ***	<0.001
Handgrip strength	30.0 ± 0.5	18.4 ± 0.4 ***	28.4 ± 0.4 *	16.5 ± 0.3 ***	<0.001
Waist circumference	81.7 ± 0.3	80.8 ± 0.5	95.0 ± 0.3 ***	93.5 ± 0.4 ***	<0.001
Body mass index	22.7 ± 0.1	22.1 ± 0.1 **	26.8 ± 0.1 ***	26.6 ± 0.2 ***	<0.001
Gender					
Male	235 (59.6)	134 (49.7)	267 (49.2)	85 (26.1)	<0.001
Female	183 (40.4)	141 (50.3)	312 (50.8)	229 (73.9)	
Marital status					
Married	414 (99.1)	274 (99.6)	575 (99.8)	309 (98.6)	0.179
Single	4 (0.9)	1 (0.4)	4 (0.2)	5 (1.4)	
Education					
Less than middle school graduate	202 (49.0)	158 (62.7)	313 (51.7)	210 (72.4)	<0.001
Middle school diploma	64 (15.0)	37 (13.6)	89 (17.2)	45 (15.7)	
High school diploma	95 (24.0)	33 (13.5)	108 (21.3)	23 (9.3)	
College degree or higher	46 (11.9)	19 (10.2)	41 (9.8)	9 (2.6)	
Employment status					
Employed	152 (36.5)	64 (23.9)	184 (29.9)	49 (15.6)	<0.001
Unemployed	254 (63.5)	183 (76.1)	369 (70.1)	238 (84.4)	
Region					
City	313 (81.8)	195 (80.5)	409 (77.3)	217 (76.4)	0.298
Rural area	105 (18.2)	80 (19.5)	170 (22.7)	97 (23.6)	
Household income					
Low	181 (41.8)	165 (56.7)	275 (44.1)	209 (66.9)	<0.001
Middle-low	126 (28.0)	59 (23.7)	154 (26.8)	66 (20.5)	
Middle-high	66 (18.9)	36 (14.2)	102 (20.0)	22 (6.9)	
High	41 (11.3)	13 (5.3)	43 (9.2)	16 (5.7)	

^1^*n* (weighted %). ^2^ *p*-value by chi square in categorical variables and general linear regression in continuous variables. ^3^ mean ± standard error. * *p* < 0.05, ** *p* < 0.01, *** *p* < 0.001 versus NSNO by LSD multiple comparisons.

**Table 2:** Clinical characteristics according to sarcopenia and obesity status.

Variables	NSNO	SNO	NSO	SO	*p*-Value ^2^
Fasting glucose (mg/dL)	133.3 ± 2.0 ^1^	133.6 ± 3.1	137.1 ± 2.2	130.3 ± 2.5	0.233
HbA_1_c (%)	7.0 ± 0.1	6.8 ± 0.1	7.1 ± 0.1	7.0 ± 0.1	0.075
Insulin (uIU/mL)	8.6 ± 0.9	10.6 ± 2.0	13.9 ± 1.1 ***	13.8 ± 2.0 *	0.002
HOMA-IR	3.0 ± 0.3	3.5 ± 0.7	4.8 ± 0.5 **	4.6 ± 0.7	0.013
Systolic BP (mm Hg)	127.8 ± 1.1	128.0 ± 1.2	129.0 ± 1.1	127.6 ± 1.5	0.822
Diastolic BP (mm Hg)	70.5 ± 0.6	67.8 ± 0.7 **	71.7 ± 0.5	69.6 ± 0.7	<0.001
Triglyceride (mg/dL)	132.4 ± 4.5	140.5 ± 6.1	164.6 ± 7.4 ***	163.7 ± 8.6 **	<0.001
Total cholesterol (mg/dL)	170.7 ± 2.3	170.1 ± 2.8	169.3 ± 1.9	171.0 ± 2.5	0.946
HDL-cholesterol (mg/dL)	46.2 ± 0.6	45.9 ± 1.0	45.2 ± 0.6	43.9 ± 0.7	0.108
LDL-cholesterol (mg/dL)	109.2 ± 5.3	103.4 ± 5.6	98.9 ± 4.6	99.6 ± 5.6	0.520

^1^ mean ± standard error. ^2^ *p*-value by chi square in categorical variables and general linear regression in continuous variables. * *p* < 0.05, ** *p* < 0.01, *** *p* < 0.001 versus NSNO by LSD multiple comparisons. HOMA-IR, homeostatic model assessment for insulin resistance; BP, blood pressure; HDL, high density lipoprotein; LDL, low density lipoprotein.

**Table 3:** Health and dietary behavioral characteristics according to sarcopenia and obesity status.

Variables	NSNO	SNO	NSO	SO	*p*-Value ^2^
Smoking					
Current smoker	45 (11.8) ^1^	33 (12.6)	54 (8.2)	23 (8.0)	0.245
Past or non-smoker	371 (88.2)	231 (87.4)	520 (91.8)	284 (92.0)	
Alcohol consumption					
No	243 (57.0)	188 (69.6)	367 (65.0)	247 (81.2)	<0.001
Yes	173 (43.0)	79 (30.4)	206 (35.0)	63 (18.8)	
Stress					
Yes	62 (14.2)	58 (19.8)	89 (15.7)	70 (24.5)	0.019
No	352 (85.8)	207 (80.2)	485 (84.3)	238 (75.5)	
Nutrition education					
Yes	38 (10.3)	23 (7.5)	60 (10.4)	29 (9.7)	<0.001
No	380 (89.7)	252 (92.5)	519 (89.6)	285 (90.3)	
Eating-out					
≥1 time/week	200 (50.6)	89 (35.1)	291 (53.9)	116 (33.2)	<0.001
1–3 times/month	138 (31.5)	92 (31.2)	176 (27.5)	107 (33.8)	
Rarely (<1 time/week)	80 (17.9)	94 (33.7)	112 (18.6)	91 (33.0)	
Dietary supplement					
Yes	229 (57.9)	119 (42.3)	287 (50.6)	138 (44.5)	0.003
No	189 (42.1)	156 (57.7)	292 (49.4)	176 (55.5)	
Physical activity ^3^					
Yes	156 (41.1)	57 (22.8)	165 (29.3)	49 (16.8)	<0.001
No	249 (58.9)	187 (77.2)	388 (70.7)	239 (83.2)	

^1^*n* (weighted %). ^2^ *p*-value by chi square in categorical variables and general linear regression in continuous variables. ^3^ Physical activity was defined as vigorous activity ≥3 times a week for ≥20 min each time or moderate activity ≥5 times a week for ≥30 min each time.

**Table 4:** Korean Healthy Eating Index (KHEI) scores according to sarcopenia and obesity.

Variables	NSNO	SNO	NSO	SO	*p*-Value ^2^
Adequacy					
Have breakfast (0–10)	9.8 ± 0.1 ^1^	9.5 ± 0.2	9.5 ± 0.1	9.3 ± 0.2 **	0.027
Mixed grains (0–5)	3.1 ± 0.1	2.7 ± 0.2	2.9 ± 0.1	2.9 ± 0.2	0.211
Total fruits (0–5)	2.7 ± 0.1	2.3 ± 0.2	2.8 ± 0.1	2.4 ± 0.2	0.060
Fresh fruits (0–5)	2.8 ± 0.2	2.5 ± 0.2	2.9 ± 0.1	2.6 ± 0.2	0.157
Total vegetables (0–5)	3.6 ± 0.1	3.2 ± 0.1 **	3.8 ± 0.1	3.4 ± 0.1	<0.001
Vegetables, excluding kimchi and pickles (0–5)	3.3 ± 0.1	2.9 ± 0.1 *	3.5 ± 0.1	3.1 ± 0.1	0.002
Meat, fish, eggs and beans (0–10)	6.7 ± 0.2	5.5 ± 0.2 ***	6.6 ± 0.2	6.1 ± 0.3	0.001
Milk and milk products (0–10)	2.5 ± 0.2	2.3 ± 0.3	2.6 ± 0.2	1.9 ± 0.3	0.218
Moderation					
% Saturated fatty acid (0–10)	8.9 ± 0.2	8.8 ± 0.2	9.2 ± 0.1	9.0 ± 0.2	0.346
Sodium (0–10)	7.9 ± 0.2	8.5 ± 0.2 **	7.7 ± 0.1	7.9 ± 0.2	<0.001
% Sweets and beverage (0–10)	9.4 ± 0.1	9.4 ± 0.1	9.2 ± 0.1	9.4 ± 0.1	0.392
Balance of energy intake					
Carbohydrate (0–5)	1.8 ± 0.1	1.7 ± 0.1	1.8 ± 0.1	1.5 ± 0.1	0.280
Fat (0–5)	2.5 ± 0.1	2.5 ± 0.2	2.6 ± 0.1	2.1 ± 0.2	0.140
Total energy (0–5)	3.3 ± 0.1	2.9 ± 0.2	3.2 ± 0.1	2.7 ± 0.2 **	0.015
Total KHEI score (0–100)	68.2 ± 0.7	64.8 ± 0.9 **	68.1 ± 0.7	64.3 ± 0.9 ***	<0.001

^1^ Mean ± standard error. ^2^ Means and *p*-value by general linear regression adjusted for gender and age. * *p* < 0.05, ** *p* < 0.01, *** *p* < 0.001 versus NSNO by LSD multiple comparisons.

**Table 5:** Energy and nutrient intake according to sarcopenia and obesity status.

Variables	NSNO	SNO	NSO	SO	*p*-Value ^2^
Energy (kcal/day)	1630.9 ± 38.1 ^1^	1464.4 ± 36.7 **	1694.0 ± 41.2	1565.2 ± 45.3	<0.001
Energy (kcal/kg body weight)	28.3 ± 0.6	26.9 ± 0.7	24.8 ± 0.7 ***	23.6 ± 0.7 ***	<0.001
Carbohydrate (g/day)	279.0 ± 6.0	254.8 ± 6.2 **	290.5 ± 6.2	272.1 ± 8.9	<0.001
Fat (g/day)	26.9 ± 1.4	24.0 ± 1.3	27.9 ± 1.6	24.7 ± 1.3	0.120
Protein (g/day)	55.4 ± 1.6	47.0 ± 1.5 ***	56.4 ± 1.6	51.5 ± 1.5	<0.001
Protein (g/kg body weight)	0.96 ± 0.03	0.86 ± 0.03 *	0.82 ± 0.02 ***	0.78 ± 0.02 ***	<0.001
Dietary fiber (g/day)	25.7 ± 0.8	21.9 ± 0.9 **	27.7 ± 1.0	23.6 ± 1.1	<0.001
% Energy from macronutrients					
Carbohydrate (%)	69.6 ± 0.7	71.2 ± 0.8	70.4 ± 0.5	71.0 ± 0.8	0.454
Fat (%)	14.4 ± 0.5	13.9 ± 0.6	13.9 ± 0.4	13.5 ± 0.6	0.727
Protein (%)	13.5 ± 0.2	12.7 ± 0.2	13.3 ± 0.2	13.3 ± 0.3	0.070
% Energy from meal					
Breakfast	27.4 ± 0.6	28.7 ± 0.6	26.9 ± 0.6	28.5 ± 0.7	0.191
Lunch	31.0 ± 0.8	31.6 ± 0.9	32.2 ± 0.7	31.5 ± 0.9	0.686
Dinner	30.1 ± 0.7	30.0 ± 0.8	28.7 ± 0.6	30.9 ± 0.8	0.121
Snack	17.2 ± 0.8	17.7 ± 1.3	18.1 ± 0.8	17.3 ± 1.0	0.836

^1^ Mean ± standard error. ^2^ Means and *p*-value by general linear regression adjusted for gender and age. * *p* < 0.05, ** *p* < 0.01, *** *p* < 0.001 versus NSNO by LSD multiple comparisons.

**Table 6:** Association of combination of dietary and physical activity factors with non-sarcopenic non-obese status in diabetic elderly.

		Model 1	Model 2	Model 3
Physical activity level	Total KHEI score			
Recommended ^2^	KHEI Q4 ^3^ (ref.)	1	1	1
	KHEI Q3	0.54 (0.28–1.02) ^1^	0.56 (0.29–1.09)	0.58 (0.29–1.15)
	KHEI Q2	0.70 (0.39–1.27)	0.73 (0.39–1.36)	0.79 (0.43–1.47)
	KHEI Q	0.57 (0.29–1.12)	0.62 (0.32–1.20)	0.69 (0.34–1.37)
Insufficient	KHEI Q4	0.44 (0.27–0.73)	0.56 (0.34–0.93)	0.60 (0.36–0.99)
	KHEI Q3	0.29 (0.17–0.48)	0.34 (0.20–0.58)	0.38 (0.22–0.66)
	KHEI Q2	0.37 (0.21–0.64)	0.47 (0.27–0.83)	0.51 (0.28–0.92)
	KHEI Q1	0.24 (0.14–0.41)	0.33 (0.19–0.57)	0.38 (0.21–0.69)
Physical activity level	Level of protein intake			
Recommended	Protein T3 ^4^ (ref.)	1	1	1
	Protein T2	0.69 (0.35–1.38)	0.77 (0.38–1.58)	0.81 (0.39–1.68)
	Protein T1	0.41 (0.22–0.77)	0.50 (0.26–0.97)	0.54 (0.27–1.06)
Insufficient	Protein T3	0.64 (0.35–1.16)	0.71 (0.37–1.33)	0.73 (0.38–1.40)
	Protein T2	0.29 (0.16–0.53)	0.39 (0.20–0.74)	0.43 (0.22–0.84)
	Protein T1	0.20 (0.11–0.35)	0.29 (0.16–0.53)	0.32 (0.17–0.61)

^1^ Odds ratio (95% confidence interval) by multiple logistic regression. Model 1: Unadjusted. Model 2: Adjusted for age and gender. Model 3: Adjusted for age, gender, education, household income, smoking, alcohol consumption, stress, eating-out and dietary supplement. ^2^ Recommended physical activity level was defined as vigorous activity ≥3 times a week for ≥20 min each time or moderate activity ≥5 times a week for ≥30 min each time. ^3^ Participants were divided into quartiles for total KHEI score (Q1 < 57, Q2 = 57–65, Q3 = 66–73, Q4 = 74–100). ^4^ Participants were divided into three groups by levels of protein intake (g/kg B.W.) (T1 < 0.8, T2 = 0.8–1.2, T3 > 1.2). ref., reference.

**Table 7:** Odd ratios for sarcopenia and obesity status according to KHEI score and level of protein intake.

		Model 1	Model 2	Model 3	Model 4
Total KHEI score					
SNO	KHEI Q4 ^2^ (ref.)	1	1	1	1
	KHEI Q3	1.50 (0.86–2.62) ^1^	1.40 (0.79–2.49)	1.41 (0.77–2.55)	1.21 (0.65–2.26)
	KHEI Q2	1.55 (0.92–2.62)	1.33 (0.78–2.30)	1.32 (0.72–2.39)	1.04 (0.56–1.93)
	KHEI Q1	2.95 (1.73–5.01)	2.38 (1.38–4.11)	1.93 (1.10–3.38)	1.41 (0.76–2.61)
NSO	KHEI Q4 (ref.)	1	1	1	1
	KHEI Q3	1.62 (1.07–2.47)	1.55 (1.01–2.36)	1.63 (1.06–2.51)	1.68 (1.07–2.63)
	KHEI Q2	1.10 (0.73–1.67)	1.03 (0.67–1.58)	1.04 (0.67–1.60)	1.10 (0.71–1.72)
	KHEI Q1	1.38 (0.88–2.15)	1.29 (0.81–2.04)	1.34 (0.84–2.13)	1.35 (0.82–2.21)
SO	KHEI Q4 (ref.)	1	1	1	1
	KHEI Q3	2.41 (1.44–4.03)	2.19 (1.28–3.74)	2.16 (1.18–3.96)	1.92 (0.99–3.69)
	KHEI Q2	1.84 (1.14–2.98)	1.88 (1.13–3.14)	1.64 (0.94–2.85)	1.42 (0.78–2.60)
	KHEI Q1	3.17 (1.87–5.40)	2.78 (1.59–4.88)	2.37 (1.27–4.41)	2.00 (1.03–3.87)
Level of protein intake					
SNO	Protein T3 ^3^ (ref.)	1	1	1	1
	Protein T2	2.17 (1.30–3.63)	2.05 (1.23–3.42)	1.63 (0.93–2.85)	1.52 (0.86–2.66)
	Protein T1	3.05 (1.87–4.97)	2.62 (1.58–4.36)	1.96 (1.11–3.47)	1.59 (0.87–2.89)
NSO	Protein T3 (ref.)	1	1	1	1
	Protein T2	1.65 (0.99–2.76)	1.60 (0.96–2.68)	1.50 (0.89–2.52)	1.44 (0.85–2.43)
	Protein T1	2.52 (1.63–3.90)	2.39 (1.53–3.73)	2.11 (1.34–3.33)	2.15 (1.34–3.46)
SO	Protein T3 (ref.)	1	1	1	1
	Protein T2	2.75 (1.55–4.89)	2.79 (1.55–5.02)	2.08 (1.13–3.82)	1.88 (1.02–3.48)
	Protein T1	5.90 (3.42–10.18)	5.43 (3.05–9.65)	3.59 (1.96–6.57)	2.99 (1.60–5.57)

^1^ Odds ratio (95% confidence interval) by multiple logistic regression. Model 1: Unadjusted. Model 2: Adjusted for physical activity. Model 3: Adjusted for physical activity, age and gender. Model 4: Adjusted for physical activity, age, gender, education, household income, smoking, alcohol consumption, stress, eating-out and dietary supplement. ^2^ Participants were divided into quartiles for total KHEI score (Q1 < 57, Q2 = 57–65, Q3 = 66–73, Q4 = 74–100). ^3^ Participants were divided into three groups by levels of protein intake (g/kg B.W.) (T1 < 0.8, T2 = 0.8–1.2, T3 > 1.2). The odds ratios can be interpreted as the risk for SNO, NSO and SO relative to NSNO. ref., reference.

## Data Availability

The KNHANES datasets used in the current study can be obtained from following link: https://knhanes.kdca.go.kr/knhanes/sub03/sub03_02_05.do (accessed on 15 December 2023).

## Supplementary Materials

The following supporting information can be downloaded at: https://www.mdpi.com/article/10.3390/nu16070964/s1, Table S1: Korean Healthy Eating Index components and standards for scoring. Reference [42] cited in Supplementary Materials.
