# Supplementary material for: Association of Combining Diet and Physical Activity on Sarcopenia and Obesity in Elderly Koreans with Diabetes
Source: Nutrients. 2024 Mar 27;16(7):964. doi: 10.3390/nu16070964 (PMC11013649; doi:10.3390/nu16070964)
Supplement: Supplementary file 1 [file nutrients-16-00964-s001.zip › nutrients-2920504-supplementary.pdf]

Supplementary Table

Table S1. Korean Healthy Eating Index components and standards for scoring

Classification (No. of Components)	Components	Score Range	Standard for Maximum Score	Standard for Minimum Score
Adequacy (8)	Have breakfast *	0–10	5–7 times/week	0 times/week
	Mixed grains intake *,†	0–5	≥0.3 serving/day	0 serving/day
	Total fruits intake †	0–5	• Men aged 19–64 years: ≥3 serving/day<br>• Men aged 65 years and overs: ≥2 serving/day<br>• Women aged 19–64 years: ≥2 serving/day<br>• Women aged 65 years and overs: ≥1 serving/day	0 serving/day
	Fresh fruits intake *,†	0–5	• Men aged 19–64 years: ≥1.5 serving/day<br>• Women aged 19–64 years: ≥1 serving/day<br>• Men aged 65 years and overs: ≥1 serving/day<br>• Women aged 65 years and over: ≥0.5 serving/day	0 serving/day
	Total vegetables intake †	0–5	• Men and women aged 19–64 years: ≥8 serving/day<br>• Men aged 65 years and overs: ≥8 serving/day<br>• Women aged 65 years and overs: ≥6 serving/day	0 serving/day
	Vegetables intake excluding Kimchi and pickled vegetables intake *,†	0–5	• Men and women aged 19–64 years: ≥5 serving/day<br>• Men aged 65 years and overs: ≥5 serving/day<br>• Women aged 65 years and overs: ≥3 serving/day	0 serving/day
	Meat, fish, eggs and beans intake †	0–10	• Men aged 19–64 years: ≥5 serving/day<br>• Women aged 19–64 years: ≥4 serving/day<br>• Men aged 65 years and overs: ≥4 serving/day<br>• Women aged 65 years and overs: ≥2.5 serving/day	0 serving/day
	Milk and milk products intake †	0–10	≥1 serving/d	0 serving/day
Moderation (3)	Percentage of energy from saturated fatty acid †,‡	0–10	≤7% of total energy intake	>10% of total energy intake
	Sodium intake †,§	0–10	≤2000 mg/day	>6500 mg/day
	Percentage of energy from sweets and beverages †	0–10	≤10% of total energy intake	>20% of total energy intake
Balance of energy intake (3)	Percentage of energy from carbohydrate †,§	0–5	55–65% of total energy intake	<50% or >75% of total energy intake
	Percentage of energy intake from fat †,‡,§	0–5	15–30% of total energy intake	<10% or >35% of total energy intake
	Energy intake †,§	0–5	75–125% of the estimated energy intake requirement (EER)	<60% or >140% of EER

* Dietary guidelines for Korean adults

† Dietary Reference Intake for Koreans 2015

‡ Recommendation criteria of WHO/FAO

§ 15 or 85 percentile value in Korean adults aged 19 years and over

From Yook et al. J Nutr Health 2015, 48, 419–428. http://dx.doi.org/10.4163/jnh.2015.48.5.419
